# Anti-fibrotic Effects of Cardiac Progenitor Cells in a 3D-Model of Human Cardiac Fibrosis

**DOI:** 10.3389/fcvm.2019.00052

**Published:** 2019-04-26

**Authors:** Tom C. L. Bracco Gartner, Janine C. Deddens, Emma A. Mol, Marina Magin Ferrer, Linda W. van Laake, Carlijn V. C. Bouten, Ali Khademhosseini, Pieter A. Doevendans, Willem J. L. Suyker, Joost P. G. Sluijter, Jesper Hjortnaes

**Affiliations:** ^1^Division Heart, and Lungs, Department of Cardiothoracic Surgery, University Medical Center Utrecht, Utrecht, Netherlands; ^2^Laboratory of Experimental Cardiology, Division Heart and Lungs, Department of Cardiology, University Medical Center Utrecht, Utrecht, Netherlands; ^3^Soft Tissue Engineering and Mechanobiology, Department of Biomedical Technology, Eindhoven University of Technology, Eindhoven, Netherlands; ^4^Regenerative Medicine Center Utrecht, University Medical Center Utrecht, Utrecht, Netherlands; ^5^Division Heart and Lungs, Department of Cardiology, University Medical Center Utrecht, Utrecht, Netherlands; ^6^Department of Cell and Chemical Biology, Leiden University Medical Center, Leiden, Netherlands; ^7^Department of Bioengineering, Department of Radiology, Department of Chemical and Biomolecular Engineering, Director of Center for Minimally Invasive Therapeutics (C-MIT), University of California, Los Angeles, Los Angeles, CA, United States; ^8^Utrecht University, Utrecht, Netherlands; ^9^Netherlands Heart Institute, Utrecht, Netherlands; ^10^Central Military Hospital, Utrecht, Netherlands

**Keywords:** cardiac fibrosis, cardiac tissue engineering, *in vitro* 3D models, cardiac progenitor cells, stem cell therapy, extracellular vesicles

## Abstract

Cardiac fibroblasts play a key role in chronic heart failure. The conversion from cardiac fibroblast to myofibroblast as a result of cardiac injury, will lead to excessive matrix deposition and a perpetuation of pro-fibrotic signaling. Cardiac cell therapy for chronic heart failure may be able to target fibroblast behavior in a paracrine fashion. However, no reliable human fibrotic tissue model exists to evaluate this potential effect of cardiac cell therapy. Using a gelatin methacryloyl hydrogel and human fetal cardiac fibroblasts (hfCF), we created a 3D *in vitro* model of human cardiac fibrosis. This model was used to study the possibility to modulate cellular fibrotic responses. Our approach demonstrated paracrine inhibitory effects of cardiac progenitor cells (CPC) on both cardiac fibroblast activation and collagen synthesis *in vitro* and revealed that continuous cross-talk between hfCF and CPC seems to be indispensable for the observed anti-fibrotic effect.

## Introduction

Chronic heart failure (CHF) is the leading cause of cardiovascular death, with a 5-year mortality rate of 50% (1). End stage heart failure is characterized by excessive collagen deposition caused by adverse cardiac remodeling. The remodeling process is suggested to be primarily mediated by cardiac fibroblasts (CF) (2–4), which are activated upon myocardial injury, undergoing a phenotypical switch to myofibroblasts. Myofibroblasts are characterized by their proliferative activity, increased contractile function as a result of alpha smooth muscle actin (α-SMA) expression, and increased extracellular matrix (ECM) production. These myofibroblasts fail to undergo apoptosis and remain constitutively active. The subsequent ongoing deposition of ECM results in perpetuation of pro-fibrotic signaling and cardiac fibrosis (5, 6). Cardiac fibrosis leads to impaired diastolic function and electrophysiological abnormalities.

Current medical treatment of CHF may slow down the progression of the disease, but does not target cardiac fibrosis (7). However, experimental treatments such as the novel angiotensin receptor-neprilysin inhibitor LCZ696, that displayed positive effects on human cardiac remodeling and increased survival in human heart failure patients (PARADIGM-HF trial), led to a marked decrease in myocardial fibrosis in a rat model (8, 9). Moreover, reverse remodeling has been observed in patients receiving mechanical circulatory support (10). These findings contribute to the notion that cardiac fibrosis may be reversible and elude to a potential therapeutic target (11, 12).

Cardiac cell therapy for chronic heart failure may also target fibroblast behavior (13). Several studies have shown positive results of cardiac progenitor cells (CPC) on cardiac function, as reflected in a lower scar mass (14, 15). CPC reduced fibroblast proliferation and attenuated pro-fibrotic signaling in a porcine model of chronic MI (16). Recently, we also observed that CPC injection could preserve end-diastolic dimensions post-MI in mice. Moreover, we noticed that measurements of regional wall motion parameters by speckle tracking analysis could reveal early changes in matrix remodeling upon CPC injection (17). The anti-fibrotic effects of CPCs seem to be paracrine in nature and seem to be mediated through exosomes, microRNAs, and endoglin (18, 19). The mechanisms of action are not fully understood however, mainly due to a lack of *in vivo* insights in matrix remodeling and the role of associated CF (20).

Cell behavior is strongly influenced by the biochemical and mechanical characteristics of the ECM environment. 3D *in vitro* models have been established to study living tissues in a more physiologically relevant environment (21). This is particularly useful when applied to the investigation of cardiac fibrosis. Conventional 2D cell culture systems cannot reliably mimic the process of cardiac fibrosis, as cardiac fibroblasts cultured in 2D will spontaneously exhibit a myofibroblast phenotype due to high substrate stiffness (5).

We have previously shown the feasibility of 3D culture platforms, in combination with rodent cardiac cells, to mimic cardiac fibrosis *in vitro* (22). However, no reliable human fibrotic tissue model exists. Therefore, this study aims to use a 3D model of human cardiac fibrosis to test the paracrine effect of CPC on fibroblast behavior.

## Methods

### Hydrogel Fabrication and Preparation

The ability to tune the mechanical properties of hydrogels, makes them attractive platforms to elucidate mechanisms involved in CF activation (22). The synthesis of gelatin methacryloyl has been described before (23). Briefly, type A gelatin from porcine skin (Sigma Aldrich) was dissolved in phosphate buffered saline (PBS; Gibco) at 60°C to obtain a 10% w/v gelatin solution. Gelatin was modified with methacryloyl groups (80%) by addition of 8 mL methacrylic anhydride to 100 mL gelatin solution at a rate of 0.5 mL/min under stirred conditions at 50°C. After that, GelMA was diluted and dialyzed against distilled water to remove salts and methacrylic acid. Finally, the solution was lyophilized and stored at −80°C until further use.

Hydrogels were prepared by radical cross-linking of solubilized GelMA in PBS (Gibco) in the presence of a photo-initiator (PI; Irgacure 2959, CIBA chemicals). In short, 1 mg of PI was dissolved in 1 mL of PBS at 80°C. Lyophilized GelMA macromer (5 or 10% w/v) was mixed with 0.1% PBS-PI and incubated for 15 min at 80°C to dissolve completely.

**Figure 1:**
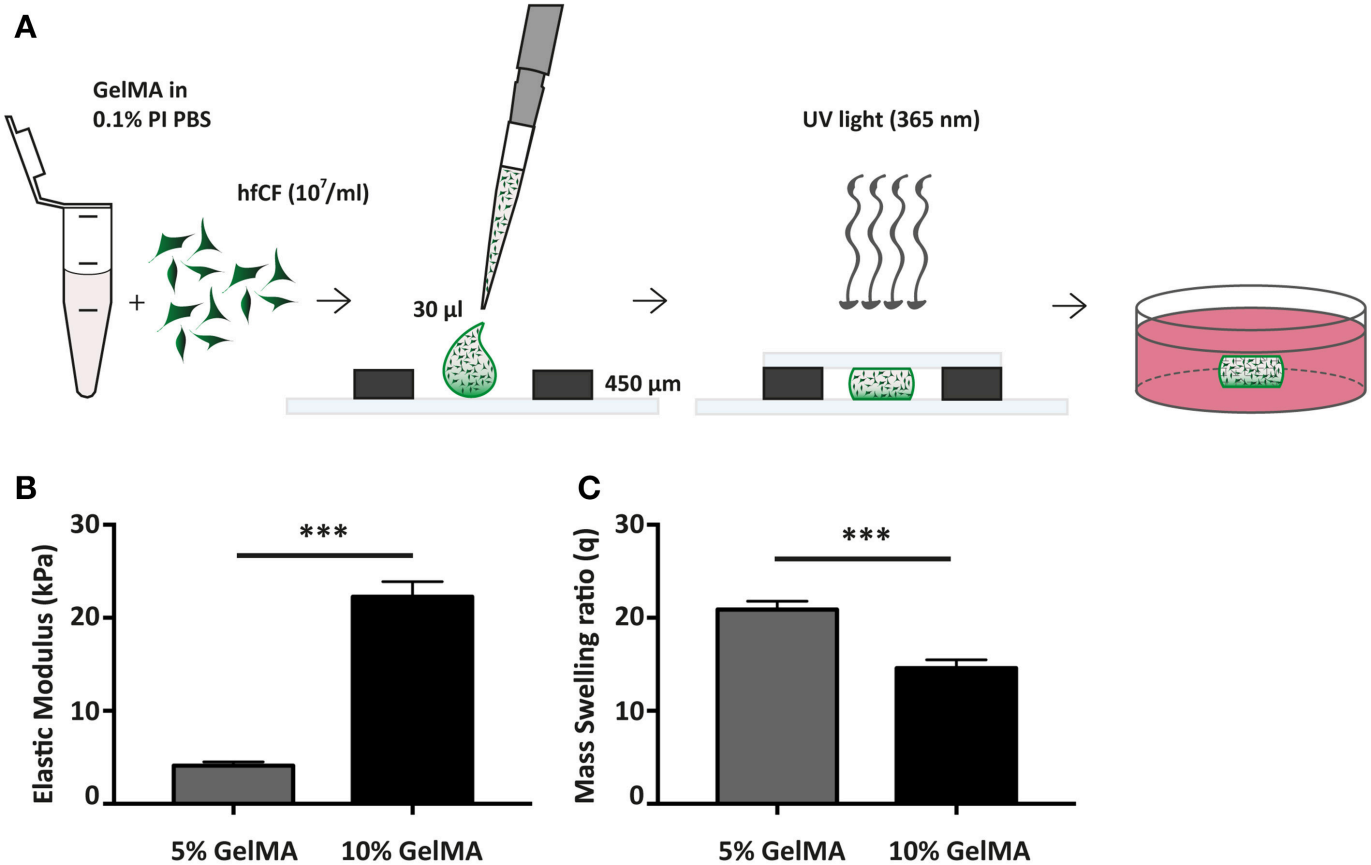
Fabrication and mechanical characterization of GelMA hydrogels. **(A)** Schematic overview of hydrogel preparation. **(B)** Compressive properties of GelMA hydrogels upon varying gel percentage (w/v), 5% GelMA (*n* = 3) and 10% GelMA (*n* = 4). Statistical significance was assessed using student's *t*-test (two-tailed). ^***^p < 0.001. **(C)** Mass Swelling ratio (q) differs with GelMA concentration. q = wet weight/dry weight. 5% GelMA (*n* = 5) and 10% GelMA (*n* = 6). Statistical significance was assessed using student's *t*-test (two-tailed). ^***^p < 0.001.

The pre-polymer solution was allowed to cool down to 37°C before either direct use or resuspension with pelleted hfCF in a final concentration of 10 million cells/mL (Figure 1A). Thirty micro liters of pre-polymer solution was pipetted on a petri dish between two spacers with a height of 0.45 mm and was covered with a sterile glass slide. The pre-polymer solution was cross-linked by exposure to UV light (wavelength 365 nm) of 2.5 W/cm^2^ at 10 cm distance for 50 s as calibrated with a R2000 UV Radiometer (Omnicure R2000 Series 2000, Excelitas Technologies®). The hydrogel was removed from the glass plate and immersed in PBS at room temperature (RT). Empty gels were incubated in PBS at 37°C for 24 h before mechanical testing and hydrogel swelling analysis. Cell-laden hydrogels were directly transferred to a non-adhesive well plate for suspension culture, containing warm culture medium.

### Hydrogel Characterization

The compressive modulus of (empty) GelMA hydrogels was assessed through a micro-indentation test, applying unconfined compression at a constant rate (0.1 mm/s) up to a strain of 70% at RT. Compressive modulus was then calculated from the linear region of the stress-strain curve. Hydrogel swelling analysis was performed directly after incubation at 37°C in PBS. Swollen GelMA hydrogels were weighed (ww) and subsequently dried by lyophilization. After that, dried weight (wd) of GelMA gels was obtained and the mass-swelling ratio (q) was calculated as q = ww/wd.

### Cell Culture

For primary cell isolation, human fetal tissue was obtained following parental permission using standard informed consent procedures. The process was approved by the ethics committees of the University Medical Center Utrecht and Leiden University Medical Center, the Netherlands. This is in accordance with the principles outlined in the Declaration of Helsinki.

Isolation of CPC was performed as described previously (24). In short, fetal cardiac tissue was enzymatically digested and cells were isolated using Sca-1 conjugated magnetic beads. CPC were cultured in SP++ (66% M199 (Gibco, 31150), 22% EGM-2 (Lonza, CC-4176), 10% fetal bovine serum (FBS) (Life-Tech, 102700), 1% Penicillin/Streptomycin [Invitrogen, 15140122), and 1% minimal essential medium nonessential amino acids (Gibco, 11140)] until 70–80% confluency and used for co-culture experiments at passage 14–18.

HfCF were isolated in a subsequently performed procedure. After subtraction of Sca-1 positive cells, the remaining dissolved heart tissue was plated overnight (o/n) on tissue culture treated plastic to allow fibroblast to adhere. HfCF were cultured in fibroblast medium (FM) containing DMEM (4,5 g/L glucose; Gibco), 10% FBS, and 1% P/S. Cells were cultured until 90% confluency and passaged in a 1:3 ratio before experimental use at passage 4–7. Cells were maintained at 5% CO_2_, 20% O_2_, 37°C, in a humidified atmosphere. To obtain conditioned medium (CM), cell cultures were maintained for 2–3 days. The CM was directly used in experiments, or used for isolation of extracellular vesicles (**Figure 5C**).

Human microvascular endothelial cells (HMEC) were cultured as before in HMEC culture medium (MCDB131, 10% FBS, hydrocortisone (50 nM, Sigma, H6909-10), human endothelial growth factor (10 ng/mL, Peprotech, 016100-15-A), freshly added L-glutamine (200 nM, Gibco, 25030-024), and 1% P/S) (25).

For co-culture experiments, hfCF-laden hydrogels were prepared as described above (day 0 = D0) and maintained in a 1:1 mixture of SP++ and FM. Starting at day 1 (D1), gels were stimulated with TGF-β_1_ (2 ng/mL; Peprotech, 100–21C) for the duration of the experiment (up to 14 days). At D0, CPC were plated at a density of 10.000 cells/cm^2^ in mixed medium. A transwell system (0.4 μm; Greiner bio-one) was used for co-culture experiments of hfCF-laden hydrogels with CPC, where hydrogels were placed in the transwells at D1 and were maintained until D7 (**Figure 4A**). Experiments investigating the priming effect of hfCF (Supplementary Figure 5) were performed by having mixed medium on hfCF-laden gels for 24 h, after which the primed medium was transferred to a 2D-culture of CPC. After 24 h on CPC, the hfCF-primed CM was transferred onto naïve hfCF-laden hydrogels. Naïve hydrogels were cultured until D7. Medium, including CM and TGF-β_1_, was changed every other day. Control experiments were performed on tissue culture plastic in two dimensions (2D), with a cell density of 10,000 cells/cm^2^.

### Isolation of Extracellular Vesicles

To isolate extracellular vesicles (EV), initially we collected serum-free CM after 24 h of CPC monoculture and EV were isolated by differential centrifugation (Beckmann Coulter LE-80K Optima), as described before (26). In short, CM was centrifuged by subsequently 2,000 x g, 10,000 x g and 100,000 x g steps. The resulting 100,000 x g pellet was resuspended in PBS and centrifuged again at 100,000 x g. The washed EV pellet was resuspended in a small volume of PBS and stored at 4°C until further use.

Subsequently, we also isolated EV using size-exclusion chromatography (SEC), as described before (26). Serum-free CM was collected after 24 h of CPC monoculture and was centrifuged at 2,000 x g to remove debris, after which it was filtered (0.45 μm). CM was then concentrated to 5 mL using 100-kDa molecular weight cut-off (MWCO) Amicon Ultra Centrifugal Filters (Merck Millipore). The concentrated CM was subsequently loaded onto a S400 highprep column (GE Healthcare, Uppsala, Sweden) using an AKTAStart (GE Healthcare) containing a 280 nm flow cell. The fractions containing EV were pooled, concentrated using 100 kDa MWCO Amicon Ultra Centrifugal Filters, and filtered (0.45 μm). The EV protein concentration was determined with the microBCA protein assay kit (Thermo Scientific). EV were stored at 4°C and added to the medium of hfCF-laden GelMA in a concentration of 3 μg/mL.

### Characterization of hfCF Encapsulated in GelMA

Viability of hfCF cells within 5 or 10% (w/v) GelMA was assessed using the Live/Dead Viability kit for mammalian cells (Life Technologies), following manufacturer instructions. In brief, hfCF-laden hydrogels (day 1, 7, 14) were quickly rinsed in PBS and then incubated with 4 μM Calcein AM and 2 μM Ethidium Homodimer-1 for 30 min at 37°C. After incubation, the gels were washed with PBS and directly imaged using a confocal microscope (Zeiss LSM 700). At three different locations in each gel, z-stacks (3.3 μm per scan) were made and the resulting 2D projection stacks were used for quantification of live (green) and dead (red) cells in Image J.

### RNA Isolation and Quantitative Real-Time Polymerase Chain Reaction

RNA was isolated from hfCF-laden GelMA hydrogels using the Nucleospin® RNA kit from Macherey-Nagel. After mechanical disruption in 1 mL TriPure (Roche), (extracellular) debris was removed by centrifugation at 12.000 x g for 10 min. Next, 200 μL of chloroform was added to the supernatant and the aqueous phase was separated by centrifugation at 12.000 x g for 15 min. The liquid phase (~350 μL) was mixed 1:1 with 70% ethanol and transferred to the isolation column after which the manufacturer's instructions were followed. Total RNA was treated with DNAse (Qiagen) and reverse transcribed with the iScript Advanced cDNA synthesis kit (Bio-RAD). Quantitative polymerase chain reaction (qPCR) was performed in iCycler qPCR (Bio-Rad) using SYBR Green (BioRad) and specific primers for GAPDH, α-SMA and collagen type 1 alpha chain 1 (COL1a1) (see Supplementary Table 1). Threshold cycle values (Ct) were analyzed and expression was quantified using the 2^−ddCt^ method (27).

### Histological Analysis and Immunofluorescent Staining of hfCF-laden Hydrogels

HfCF-laden hydrogels were washed in PBS and fixed in 4% paraformaldehyde for 25 min. Before embedding in OCT (Tissue Tek), hydrogels were partly dehydrated in a 30% (w/v) sucrose solution overnight at 4°C. Cryosections of 7 μm were obtained and all images were taken with a fluorescent microscope system BX53 (Olympus).

Immunofluorescent staining for α-SMA and Col1a1 was performed using Alexa fluor 488 labeled anti-human α-SMA (A2547, 1:400, Sigma-Aldrich) and anti-pro-collagen type 1 (SP1.D8, 1:50, DSHB), followed by incubation with an Alexa fluor 555 (Invitrogen, 1:1000) labeled secondary antibody. Sections were counterstained with Hoechst (33342). Images were taken with the aforementioned microscope and analyzed using ImageJ software. A negative control was used to determine a threshold value for Col1a1. All cells with signals above this threshold value were regarded as highly collagen positive and quantified accordingly.

Seeded and empty GelMA hydrogels with different macromere concentrations were stained with picrosirius red (Sigma Aldrich) to analyze the collagen composition of the matrix as measured by polarized light. Analysis of the images was performed using ImageJ software. Collagen density is expressed as mean gray value, adjusted for area fraction (28).

### Statistical Analysis

**Figure 2:**
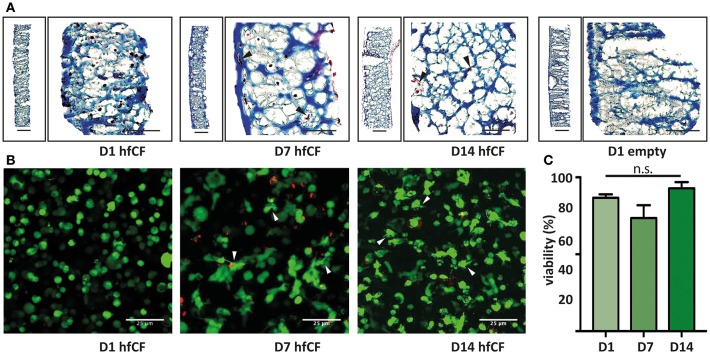
Encapsulation of human fetal cardiac fibroblasts in GelMA hydrogel. **(A)** Trichrome blue staining demonstrates homogenous encapsulation of hfCF in the 10% GelMA hydrogel. Blue = extracellular matrix and scaffold (GelMA). Dark red = hfCF. **(B)** Live/Dead staining shows that cells remain viable in the 10% GelMA hydrogels until 14 days in culture. Directly after encapsulation, cells display a round morphology, whereas after 7 days cells appear more elongated and attached to the matrix (arrowheads). Live cells = green. Dead cells = red. Scale bars = 25 μm. **(C)** Semi-quantitative analysis of the number of live cells (*n* = 5). Statistical significance was assessed using one-way ANOVA.

**Figure 3:**
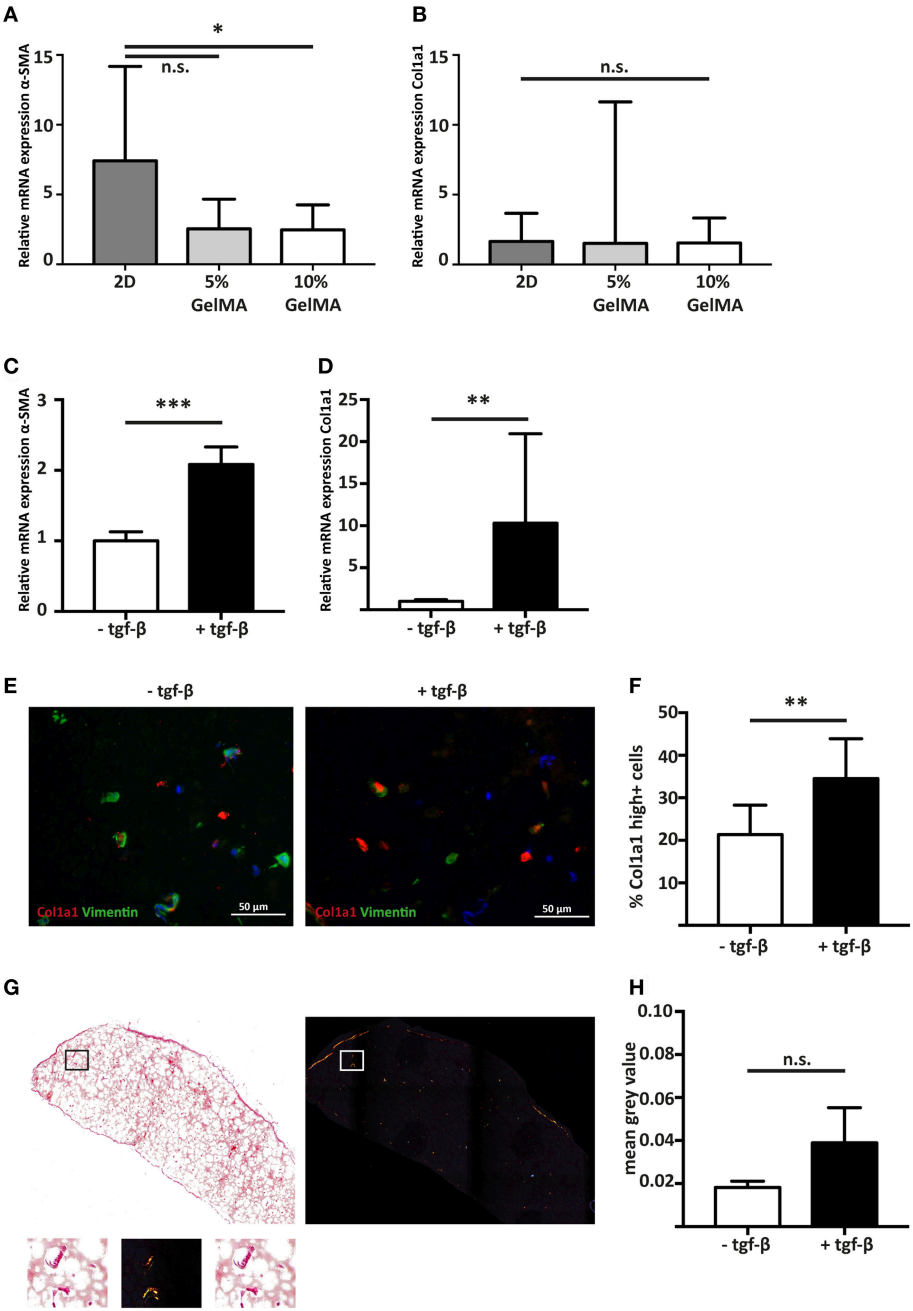
Stimulating cardiac fibroblast mediated fibrosis with the pro-fibrotic mediator TGF-β1. **(A)** Gene expression analysis shows that hfCF have a lower α-SMA expression after 7 days of culture when encapsulated in 5% (w/v) or 10% (w/v) GelMA (*n* = 8). Statistical significance was assessed using one-way ANOVA with a Dunnett *post-hoc* test. ^*^p < 0.05. **(B)** Col1a1 expression remains equal. Values are relative to the corresponding 2D cultured hfCF at day 1 (*n* = 8). Statistical significance was assessed using one-way ANOVA. **(C)** Stimulation with TGF-β1 for 7 days results in a 2.1-fold increase in α-SMA expression and **(D)** a 10.3-fold increase in Col1a1 expression (*n* = 8). Statistical significance was assessed using paired student's *t*-test (two-tailed). ^***^p < 0.001 ^**^p < 0.01. **(E,F)** Immunohistochemistry shows an increased number of highly Col1a1 positive cells upon stimulation with TGF-β1 (Col1a1 = red, vimentin = green, nuclei = blue). Statistical significance was assessed using paired student's *t*-test (two-tailed). ^**^p < 0.01. **(G,H)** Accumulation of collagen fibers in the extracellular matrix is demonstrated by picrosirius red staining and quantification (*n* = 4). Left panel = bright field image. Right panel = polarized light image. Lower panel shows a higher magnification of the box. Statistical significance was assessed using paired student's *t*-test (two-tailed).

**Figure 4:**
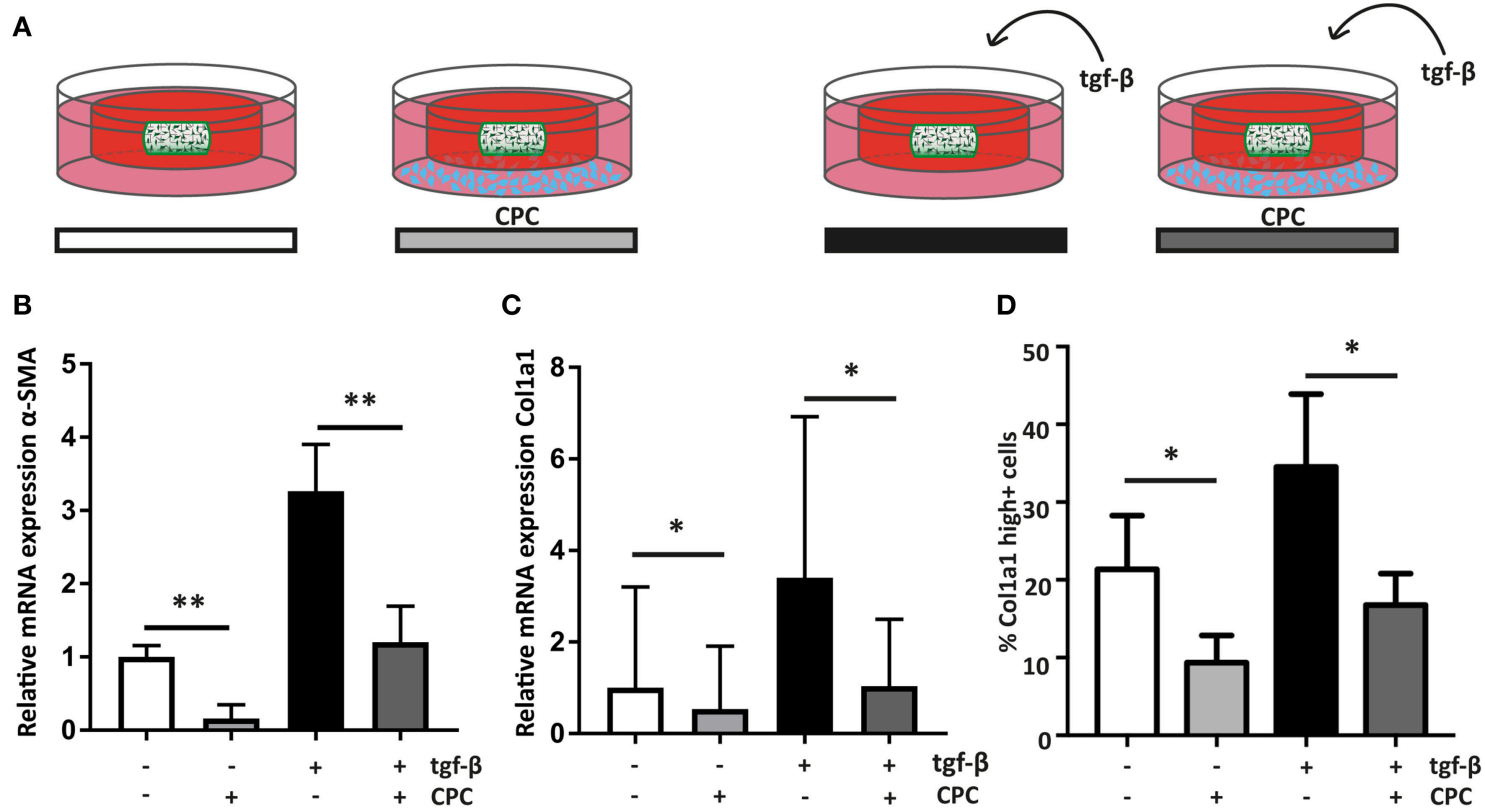
Cardiac progenitor cells attenuate the cardiac fibroblast mediated fibrotic response. **(A)** Schematic overview of the experimental set-up. **(B)** CPC attenuate α-SMA expression in hfCF-laden GelMA in culture conditions with or without 2 ng/ml TGF-β_1_ stimulation (*n* = 13). **(C)** Col1a1 expression is lower in co-culture conditions (*n* = 12) and **(D)** likewise, the amount of highly Col1a1 positive cells is 2-fold lower in CPC-treated gels (*n* = 4). Statistical significance was assessed using repeated measures two-way ANOVA. ^**^p < 0.01 ^*^p < 0.05.

**Figure 5:**
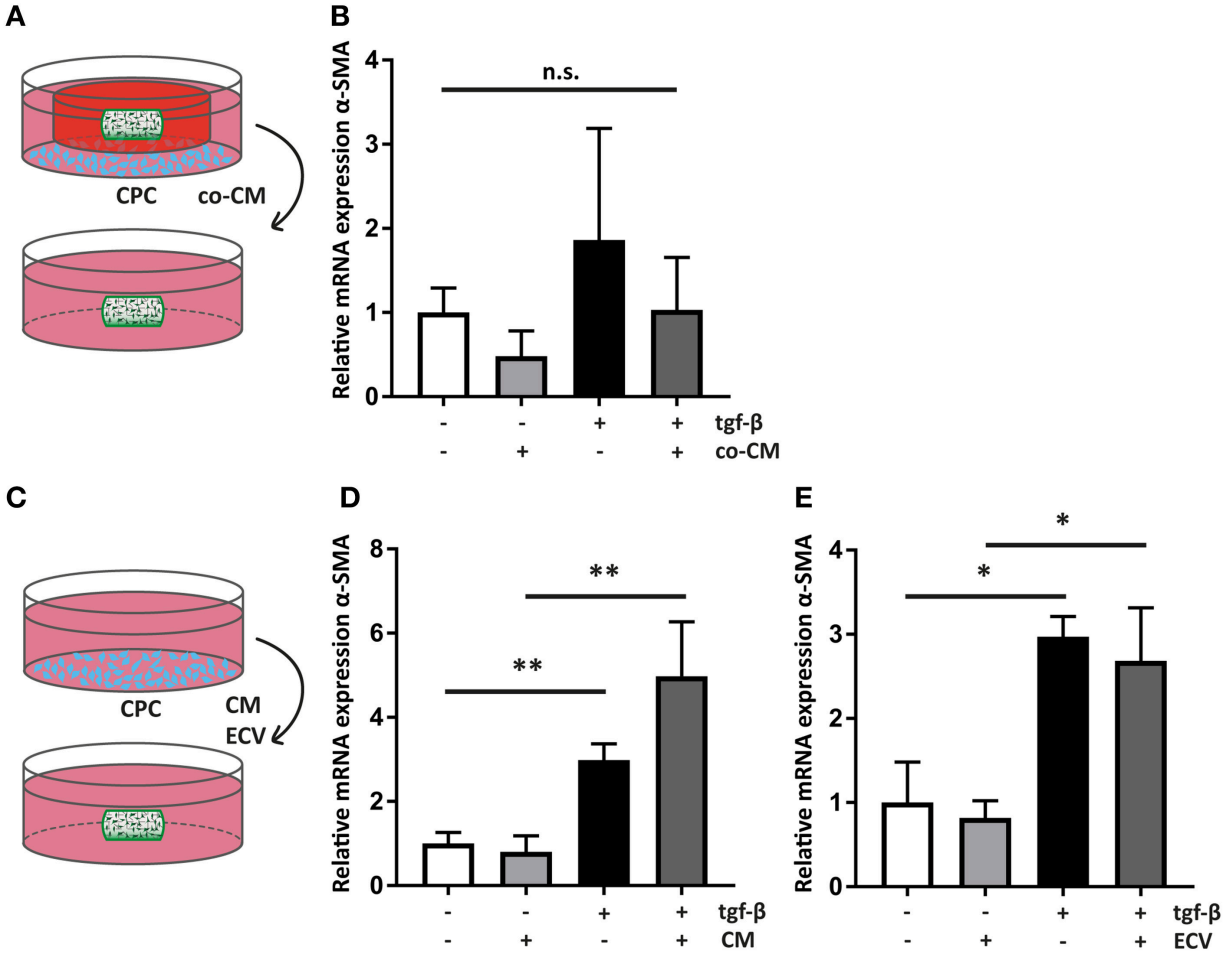
Cardiac progenitor cells act via paracrine mediators. **(A)** Schematic overview of the experimental set-up of transfer of co-CM to a naïve hfCF-laden hydrogel. **(B)** co-CM attenuates α-SMA expression in hfCF-laden GelMA in culture conditions with or without 2 ng/ml TGF-β_1_ (*n* = 5). Statistical significance was assessed using repeated measures two-way ANOVA. **(C)** Schematic overview of the experimental set-up of treatment of naïve hfCF-laden hydrogels with CPC conditioned medium (CM) or CPC-derived extracellular vesicles (EV). **(D)** CM does not influence hfCF activation. In pro-fibrotic medium the expression of α-SMA is significantly increased (*n* = 8). Statistical significance was assessed using repeated measures two-way ANOVA. ^**^p < 0.01. **(E)** Isolated EV show similar effects as CM (*n* = 3). Statistical significance was assessed using repeated measures two-way ANOVA. ^*^p < 0.05.

Statistical analysis was performed using SPSS (IBM SPSS Statistics for Windows, Version 23.0, Chicago) and graphs were made using GraphPad Prism 7.02 (Graphpad Software, La Jolla). Differences between two groups were analyzed using paired two-tailed student's *t*-tests. Differences between multiple groups were analyzed using one-way ANOVA with a Dunnett-test *post-hoc* (in Figures 2, 3A,B and Supplementary Figure 2). Two-way ANOVA for repeated measures was used to evaluate differences between multiple groups (in Figures 4, 5 and Supplementary Figure 3). *P* < 0.05 was considered statistically significant. Data is presented as mean ± SEM, unless indicated otherwise.

## Results

### Cells Isolated From Fetal Hearts Express Fibroblast Markers and Are Viable When Cultured in a 3D GelMA Hydrogel

In this study, we aimed to construct a human model of cardiac fibrosis using hfCF and GelMA (Figure 1A). hfCF were isolated by enzymatic digestion of human fetal cardiac tissue. Immunofluorescent staining confirmed the fibroblast-like phenotype of hfCF as shown by protein expression of vimentin and fibroblast specific protein (FSP). The low positivity for FSP in our cell lines confirms previous reports on low FSP expression in cardiac fibroblasts (29, 30). In addition, hfCF expressed Col1a1, Col3a1, and α-SMA (Supplementary Figures 1A,B).

Since 5 and 10% GelMA (w/v) hydrogels have previously been demonstrated to be compatible with both rat cells (22) and cells of mesenchymal origin (31), we have determined the mechanical properties of both preparations (Figure 1B). Mechanical analysis demonstrated an elastic modulus of 4.1 ± 0.4 kPa for 5% GelMA and 22.3 ± 1.6 kPa for 10% GelMA (*p* < 0.001). In addition, the mass swelling ratio (q), which is proportional to pore size, was determined and showed a higher q for 5% than for 10% GelMA (20.9 ± 0.9 vs. 14.6 ± 0.9, *p* < 0.001; Figure 1C).

Subsequently, hfCF were encapsulated in GelMA and the biocompatibility was examined by measuring cell spreading and viability. After encapsulation, hfCF were homogeneously distributed throughout the GelMA hydrogel (Figure 2A) and showed an average viability of 89% in 5% GelMA (Supplementary Figures 2A,B) and 85% in 10% GelMA over all time points (Figures 2B,C). By day 7 of culture, hfCF encapsulated in 5% GelMA had an elongated phenotype as visualized by live/dead staining. In contrast, a rounder cell-phenotype was observed for hfCF encapsulated in 10% GelMA.

### Cardiac Fibroblasts Remain Quiescent in 3D Culture, but Exhibit a Fibrotic Response After Stimulation With TFG-β_1_

To determine the influence of a 3D culture environment, the expression of α-SMA and Col1a1 of encapsulated hfCF was analyzed and compared to 2D cultured hfCF. Conventional 2D culture resulted in a higher α-SMA expression (7.4 ± 6.8) then 3D culture in 5% (2.5 ± 2.1, *p* = 0.06) or 10% GelMA (2.5 ± 1.8, *p* = 0.05, Figure 3A). In contrast, no difference was observed for Col1a1 in 2D culture (1.7 ± 2) vs. 3D culture in 5% GelMA (1.5 ± 10.1) and 10% GelMA (1.5 ± 1.8, Figure 3B).

Since 10% GelMA hydrogels demonstrated a more accurate simulation of the mechanical characteristics of the myocardial tissue (32), we used 10% GelMA for our follow-up experiments. To examine the potential of hfCF-laden 10% GelMA as a model for human cardiac fibrosis, hfCF-laden hydrogels were cultured for 7 days in medium containing 2 ng/mL TFG-β_1_. Upon stimulation with TFG-β_1_, the expression of α-SMA increased 2.1-fold (*p* < 0.001, Figure 3C). In addition, the expression of Col1a1 increased 10.3 times (*p* < 0.01, Figure 3D), which was reflected in a higher number of Col1a1-positive cells (21 ± 7 vs. 35 ± 9%, *p* = 0.01, Figures 3E,F). Picrosirius red staining showed a trend toward accumulation of deposited collagen fibers upon TFG-β_1_ stimulation (mean gray value 0.02 ± 0.003 vs. 0.04 ± 0.016, *p* = 0.1, Figures 3G,H).

### Co-culture With CPC Decreases the Fibrotic Response in hfCF-Laden Hydrogels

To analyze if CPC are able to inhibit the observed fibrotic response via paracrine signaling, hfCF-laden GelMA were indirectly co-cultured with CPC using a transwell system (Figure 4A). After 7 days of co-culture, α-SMA expression in hfCF was decreased in the presence of CPC. Stimulation with TGF-β_1_ resulted in an increased α-SMA expression, whereas in co-culture with CPC this increase was not observed (Figure 4B). Co-culture with CPC resulted in a 2.7-fold lower α-SMA expression (*p* = 0.009, Figure 4B) and a 3.3-fold lower Col1a1 expression (*p* = 0.037, Figure 4C). This inhibitory effect was not observed in a similar set-up with HMEC, which served as non-cardiac, non-progenitor control cells (Supplementary Figure 3). Interestingly, a similar effect was seen in conditions without TFG-β_1_ stimulation; α-SMA expression decreased 6.25-fold (*p* = 0.009). In addition, this decrease in activity of hfCF in the presence of CPC was also demonstrated by the amount of Col1a1-positive hfCF in the hydrogels (Figure 4D), which showed a significant 2-fold decrease in co-culture conditions (*p* = 0.02). Collagen 3 (Col3) expression levels were also analyzed using qPCR but did not reveal significant differences upon TGF-β_1_ stimulation or co-culture with CPC. The resulting Col1/Col3 ratio therefore showed an increase upon TGF-β_1_ stimulation and a normalization upon co-culture with CPC, but these changes were not statistically significant (Supplementary Figure 4).

### Paracrine Anti-fibrotic Effects of CPC Are Transferable

All co-culture experiments were performed using transwell systems with a pore size of 0.4 μm, preventing direct cell-cell interactions between hfCF-laden GelMA hydrogels and CPC. Therefore, the observed inhibition of hfCF by CPC should be considered a paracrine effect. To confirm this, co-CM was added to naïve hfCF-laden GelMA (Figure 5A). Expression of α-SMA was measured, which showed that co-CM caused a similar inhibition of the fibrotic response as seen in previous experiments, although the differences between groups only displayed a trend toward significance (Figure 5B).

Since an inhibitory effect occurred in the non-stimulated conditions as well, we hypothesized that CM of CPC monoculture could result in a similar outcome. Therefore, CM was collected and was added to naïve hfCF-laden hydrogels (Figure 5C). However, CM did not result in a similar inhibitory effect on cardiac fibroblast activation. On the contrary, it caused a 1.7-fold increase in α-SMA expression upon stimulation with TFG-β_1_ (*p* = ns; Figure 5D).

Recently, extracellular vesicles of CPC have been shown to be important anti-fibrotic mediators (33–35). To further examine their anti-fibrotic properties, EV were isolated from CM and applied to hfCF-laden GelMA (Figure 5C). This supplementation of EV did not result in any significant changes in hfCF activation in both TFG-β_1_ unstimulated and stimulated conditions (Figure 5E).

Subsequently, we hypothesized that priming CPC with conditioned medium derived from hfCF monoculture could lead to CPC monocultures expressing anti-fibrotic mediators. Exposing naïve hfCF-laden hydrogels to hfCF-primed CM showed a 1.3-fold increase in α-SMA expression upon stimulation with TFG-β_1_ (*p* = ns; Supplementary Figure 5).

## Discussion

This study demonstrated that a 3D hydrogel culture platform of hfCF can be used to simulate human cardiac fibrosis. Encapsulating hfCF in 10% GelMA resulted in a quiescent cell phenotype. Subsequent stimulation of hfCF-laden GelMA with TFG-β_1_ resulted in myofibroblast activation and accumulation of ECM. By using this tunable human cardiac fibrosis model, we were able to investigate the effect of CPC on the fibrotic process *in vitro*. CPC inhibited the induced fibrotic response, which we were now able to demonstrate in a human *in vitro* setting, confirming previous *in vivo* findings for CPC and *in vitro* findings for cardiosphere-derived cells (CDC) (33, 36, 37). Interestingly, both non-co-cultured CM and EV did not have an anti-fibrotic effect. It seems that the anti-fibrotic action of CPC is only established after continuous crosstalk with hfCF and is probably not an action that CPC exert individually.

GelMA has been well established as an ECM substitute with tunable mechanical properties, combined with cell binding sites and the possibility of proteolytic degradation. By changing the GelMA concentration, the degree of functionalization, the photo-initiator concentration or the UV exposure time, 3D environments with different elastic moduli and pore sizes can be generated (23, 38).

Mechanical stiffness of the culture substrate is directly related to the differentiation of cultured cells (39). This is also the case for CF, as demonstrated by studies using substrates with a tunable stiffness (40–43). The elastic modulus of the healthy heart is around 18 kPa (44). Our results showed that hfCF were more quiescent when cultured in a 3D environment with heart-like elasticity, which have a much lower matrix stiffness than tissue culture plates. Similarly, earlier studies showed that fibroblasts-like valvular interstitial cells can be kept quiescent in 3D tissue constructs (45). Yet, another study demonstrated that valvular interstitial cells encapsulated in soft 5% GelMA spontaneously differentiate toward a myofibroblast phenotype (31). We did not find an increased activation in our hfCF-laden 5% GelMA as demonstrated by a low α-SMA expression. However, we did see more elongated cells in this condition, which generally indicates a higher actin expression, possibly caused by another actin isoform. This discrepancy can otherwise be explained by the different origin of these fibroblasts, the duration of the experiment or slight differences in the degree of crosslinking (46).

Tissue engineered models of cardiac fibrosis are indispensable for advancements in the field of anti-fibrotic therapeutics and have already been applied to study the mechanism of cardiac cell therapy. Previously, a 3D fibrosis model has been used to illuminate the role of mesenchymal stem cells (MSC) on myofibroblast transition and it showed an inhibitory effect of MSC on the fibrotic response in a rat model (47). Likewise, cardiospheres were shown to downregulate the TGF-β pathway in rat cardiac fibroblasts in a paracrine fashion, mediated by soluble endoglin (36, 48). Also, the anti-fibrotic properties of human adipose-derived stem cells were confirmed *in vitro* using a murine cardiac fibrosis model. These stem cells inhibit myofibroblast differentiation by secretion of hepatocyte growth factor (HGF), which causes downregulation of the angiotensin II type 1 receptor, which in turn results in downregulation of TGF-β_1_ (43). These studies support the paracrine hypothesis postulating that stem cells exert their actions via paracrine mediators (20, 49).

Based on these observations it can be hypothesized that CPC interfere with the TGF-β cascade. However, broad targeting of TGF-β activities appears not to be beneficial (11) and is challenged by the miscellaneous functions of the TGF-β pathway and its dual role in both activation and inhibition of the fibrogenic response, via ALK5 and ALK1, respectively (50). Although we observed an attenuation of the fibrotic response upon CPC treatment, we also noticed that in the groups treated with CPC there was still an increase in hfCF activation due to the addition of exogenous TGF-β. This finding supports the idea that CPC probably modulate the TGF-β pathway, thereby tuning instead of inhibiting it.

We further investigated the anti-fibrotic effect of CPC by examining co-CM, CM, and EV. As expected, the use of co-CM resulted in a similar attenuation of the fibrotic response as seen in the preceding co-culture experiment, albeit somewhat less pronounced. This could indicate that the responsible paracrine mediators have short half-lives. On the contrary, CM and EV derived from a monoculture condition did not have an anti-fibrotic effect, nor did hfCF-primed CM. Apparently, the secretion of CPC changes upon continuous co-culture with hfCF.

Our study shows that co-culture conditions seem to be important for the anti-fibrotic effect of CPC. Several other studies have shown an anti-fibrotic effect by using MSC on murine CF, both in co-culture conditions and using conditioned medium derived from MSC monoculture (43, 51–53). The discrepancy with our results could be explained by the lack of 3D culture systems in those studies, the elastic modulus of the culture substrate or the different origins of the cell sources, in which interspecies differences might play a role.

The beneficial effects of CPC co-culture in this study seem evident. Other groups have recently shown that these effects are mediated by extracellular vesicles (33–35). Ideally, we would have isolated extracellular vesicles from co-CM and subsequently sorted them by cellular origin. This would enable us to explore the anti-fibrotic properties of EV released by CPC in co-culture conditions and facilitate a comparison with EV derived from CPC monoculture. Unfortunately, currently there are no means to distinguish different extracellular vesicle populations derived from the different cell types included (54, 55). Subsequent identification of the responsible EV-related inhibitors of fibrosis could have significant therapeutic potential. Focusing on the inhibitory effects of EV might open new doors for cell treatment in heart failure patients, particularly considering their advantage as non-living carriers and therefore better manageability as an off-the-shelf therapeutic (54).

Although the cardiac fibrosis model in our study has taken several important variables into account while designing the experiments—such as elastic modulus, 3D environment, and a relevant cell source—it is not a complete recapitulation of the human situation. Even though we chose an elastic modulus similar to the native myocardium, this elastic modulus might have changed during the experiments. Degradation of the extracellular matrix due to proteases in the serum or the remodeling actions of the fibroblasts might alter the elastic modulus (38, 56). Earlier work of our group showed that TGF-β_1_ stimulation increased the elastic modulus of GelMA hydrogels in which murine cardiac fibroblasts and cardiomyocytes were embedded (22). Changes in elastic modulus can subsequently affect cells again through mechanosensitive pathways.

Furthermore, to capture the full process of cardiac fibrosis, it is important to incorporate cardiomyocytes and vascularization in our models. The interactions between various cell types are crucial for cardiac homeostasis (57). To that end, we have previously shown that induction of fibrosis in a co-culture system of rat cardiomyocytes and CF resulted in mechanical alterations and asynchronous beating of the tissue constructs (22). Another study showed a decrease in mouse cardiomyocyte contraction force following an increase in fibroblast density in co-culture conditions. At higher fibroblast densities also the beating frequency of the cardiomyocytes decreased (58). In short, to advance cardiac fibrosis research it is indispensable to use the appropriate cell populations and extracellular matrix materials in order to mimic the native heart.

Finally, many of the pathways involved in cardiac fibroblast activation are mechanosensitive (59). The convergence of biochemical and mechanical signals is an important, but not extensively studied aspect of cardiac fibrosis. Recently however, cardiomyocyte stretch has been observed to have a pro-fibrotic effect on fibroblast proliferation in a 2D co-culture model (60). This indicates that in the fibrotic process it is not only the mechanosensitivity of fibroblasts that has to be investigated. Future research into cardiac fibrosis should take the mechanobiology of both cardiac fibroblasts and other cell types in the heart into account.

In summary, our data suggest that hfCF-laden GelMA is a suitable model to mimic human cardiac fibrosis, thereby providing a platform for pathophysiological studies and drug testing. Using this model, our study was able to demonstrate the anti-fibrotic effects of CPC *in vitro*, also revealing an important role for continuous cross-talk between CPC and hfCF. Our study emphasizes the potential of cardiac fibrosis as a therapeutic target.

## Ethics Statement

This study was carried out in accordance with the recommendations of local ethics committees with written informed consent from all subjects. All subjects gave written informed consent in accordance with the Declaration of Helsinki. The protocol was approved by the ethics committees of the University Medical Center Utrecht and Leiden University Medical Center, the Netherlands.

## Author Contributions

Experiments were performed by TB, JD, EM, and MM. TB and JD interpreted data, did statistical analysis, and wrote the manuscript. LvL, CB, AK, PD, WS, JS, and JH edited the manuscript. All authors have read and agreed to the content of the manuscript.

### Conflict of Interest Statement

The authors declare that the research was conducted in the absence of any commercial or financial relationships that could be construed as a potential conflict of interest.
