# Supplementary material for: Anti-fibrotic Effects of Cardiac Progenitor Cells in a 3D-Model of Human Cardiac Fibrosis
Source: Front Cardiovasc Med. 2019 Apr 26;6:52. doi: 10.3389/fcvm.2019.00052 (PMC6497755; doi:10.3389/fcvm.2019.00052)
Supplement: Supplementary file 1 [file Data_Sheet_1.docx]

Supplementary Material

Anti-fibrotic effects of cardiac progenitor cells in a 3D-model of human cardiac fibrosis.

Tom C.L. Bracco Gartner, Janine C. Deddens, Emma A. Mol, Marina Magin Ferrer, Linda W. van Laake, Carlijn V.C. Bouten, Ali Khademhosseini, Pieter A. Doevendans, Willem J.L. Suyker, Joost P.G. Sluijter*, Jesper Hjortnaes*

*** Correspondence:** Jesper Hjortnaes: [jhjortna@umcutrecht.nl](mailto:jhjortna@umcutrecht.nl)

Joost P.G. Sluijter: [j.sluijter@umcutrecht.nl](mailto:j.sluijter@umcutrecht.nl)

**
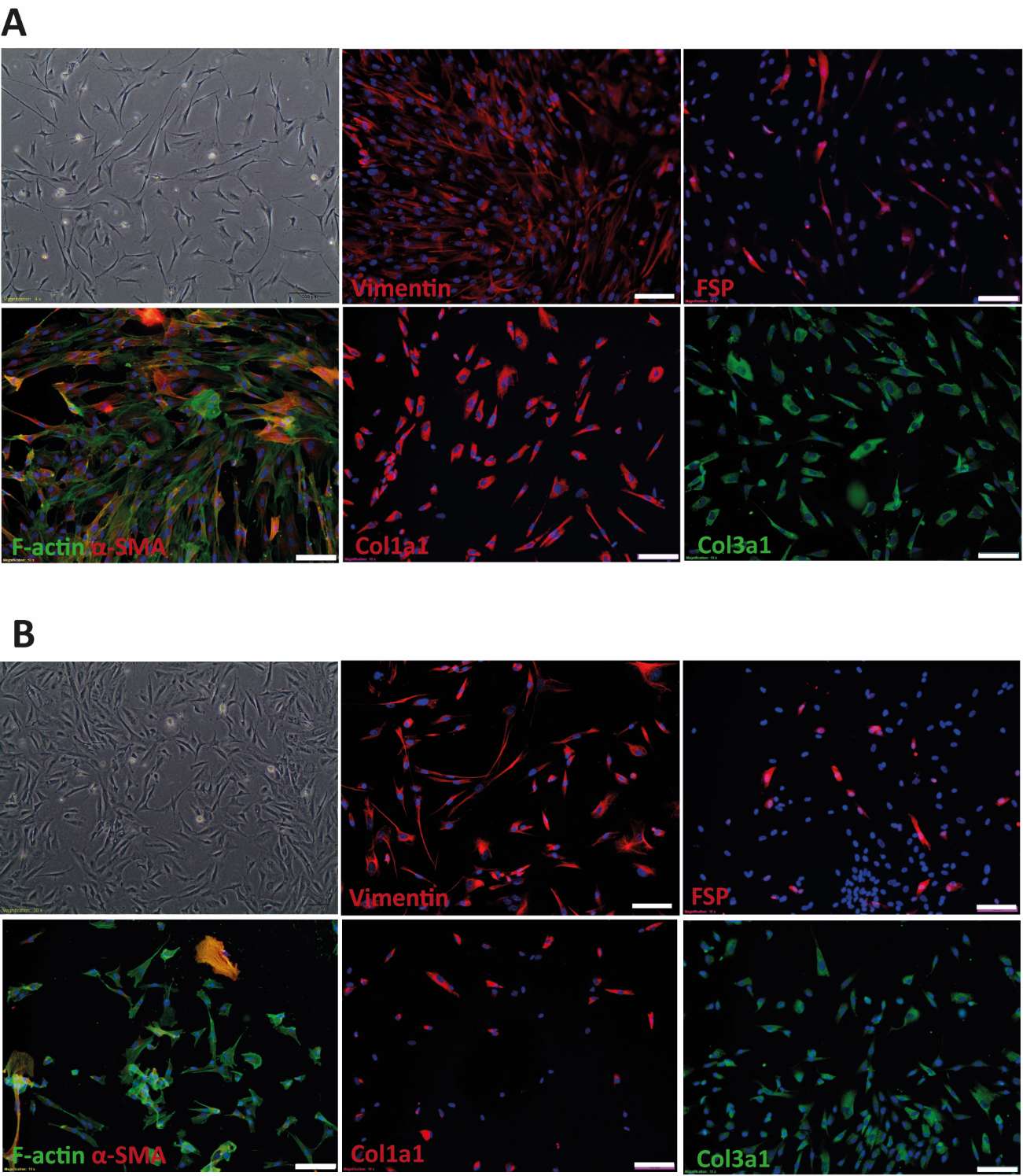
**

**Supplementary Figure 1. Immunofluorescent stainings of human fetal cardiac fibroblasts.**

hfCF were isolated from different donor hearts (**A** and **B**) and stain positive for the mesenchymal marker vimentin and partly positive for fibroblast specific protein (FSP). They also express collagen type 1a1 (Col1a1), collagen type 3a1 (Col3a1) and alpha smooth muscle actin (α-SMA). Nuclei were stained with Hoechst (blue). Scale bar = 100 μm.

**
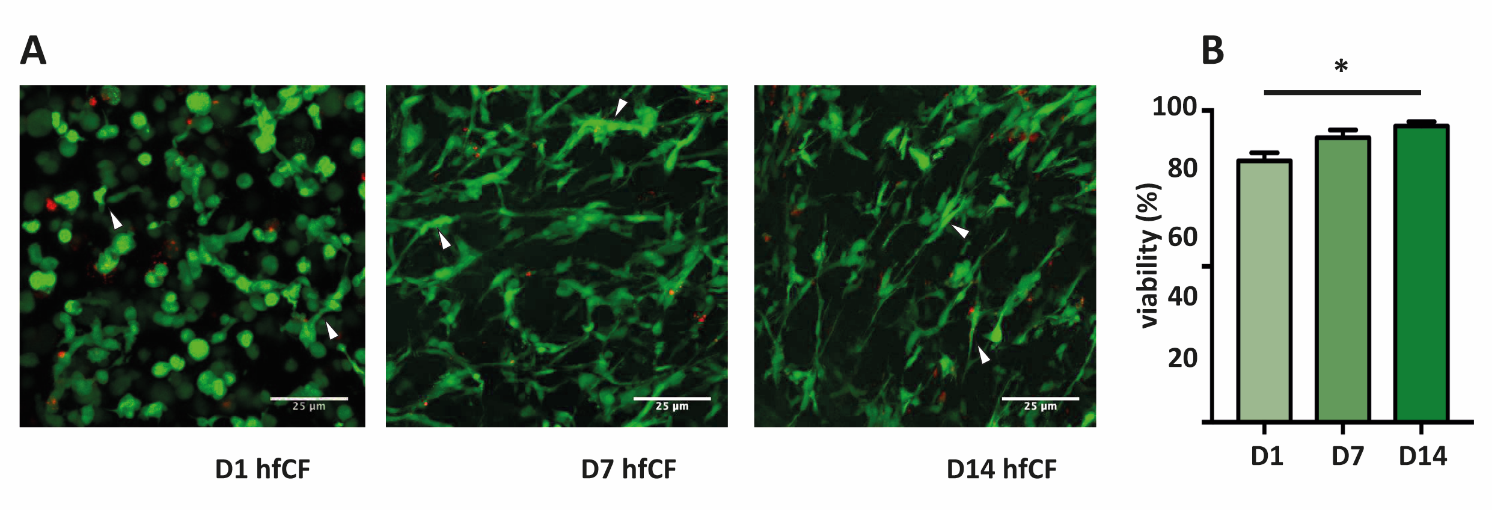
**

**Supplementary Figure 2. Viability of hfCF in 5% GelMA.**

**A)** Live/Dead staining shows that cells have a high viability (>85%) in the 5% GelMA hydrogels. Cells appear attached to the matrix at day 1 and display an elongated phenotype at D7 and D14 (white arrowheads). Live cells = green. Dead cells = red. Scale bars = 25 μm. **B)** Semi-quantitative analysis of the number of live cells (n=5). Statistical significance was assessed using one-way ANOVA. *p < 0.05.

**
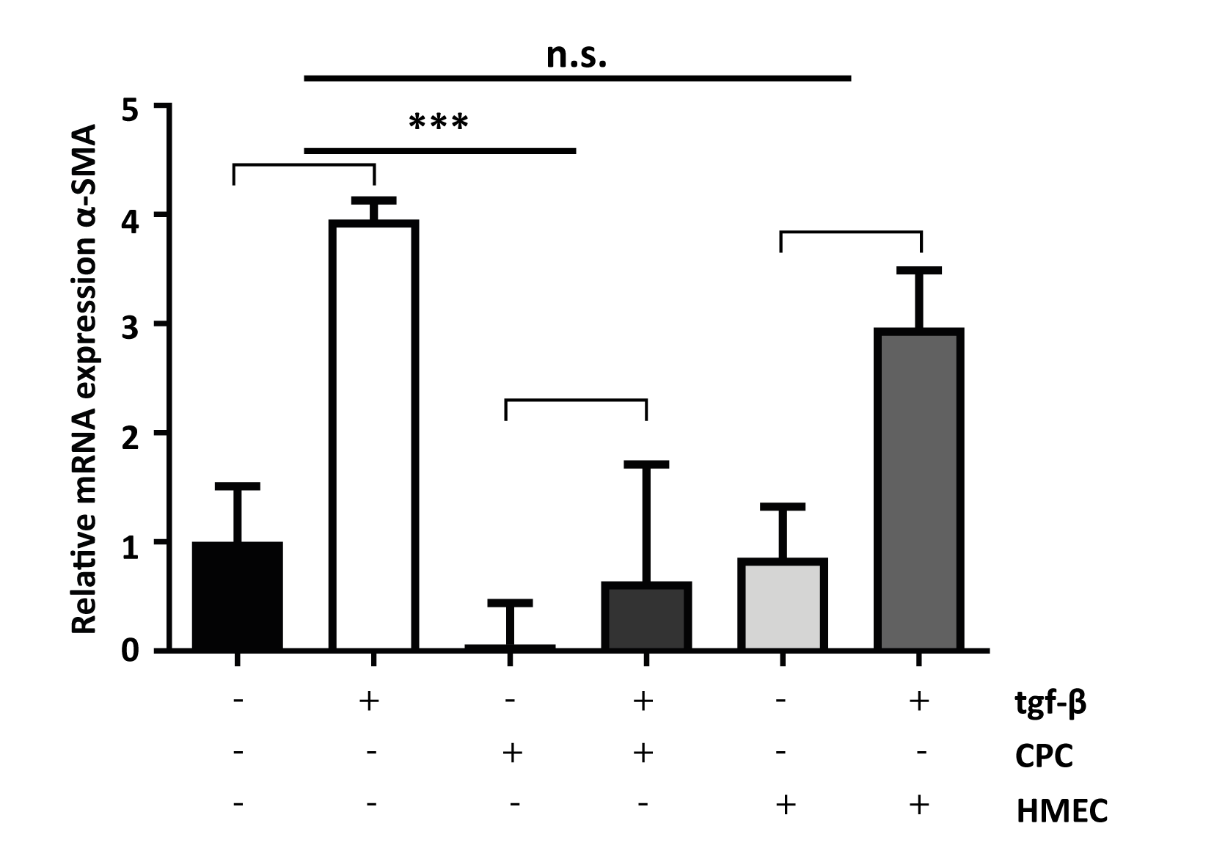
**

**Supplementary Figure 3. Inhibitory effect of CPC is cell-specific.**

hfCF-laden GelMA hydrogels were stimulated with TGF-β_1_ (2 ng/mL) and co-cultured for 7 days with either CPC or HMEC (n=4). α-SMA expression is inhibited upon co-culture with CPC, but not upon co-culture with HMEC. Statistical significance was assessed using repeated measures two-way ANOVA. ***p < 0.001.


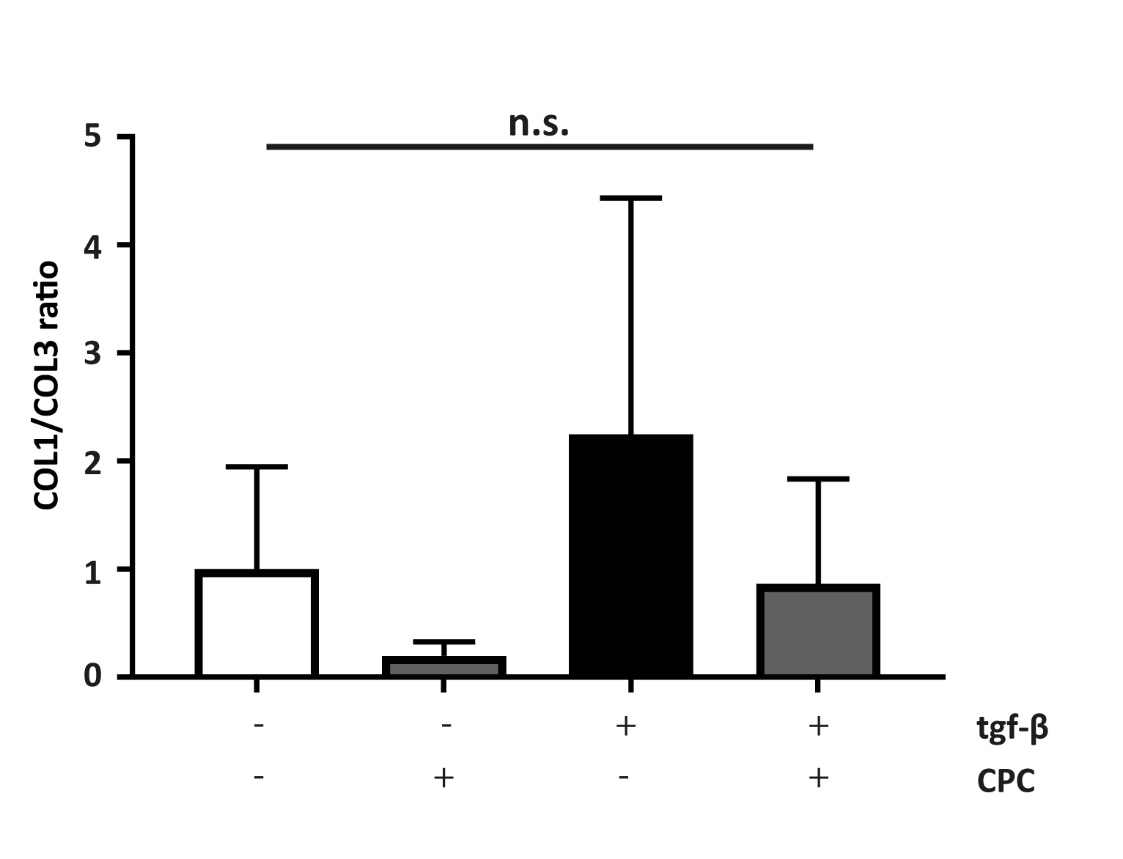


**Supplementary Figure 4. Effect of cardiac progenitor cells on Collagen1/Collagen3-ratio.**

hfCF-laden GelMA hydrogels were stimulated with TGF-β_1_ (2 ng/mL) and co-cultured for 7 days with CPC (n=3). Collagen 1 and Collagen 3 expression were quantified using qPCR and the ratio was calculated. Collagen1/Collagen3-ratio did not differ significantly between groups. Statistical significance was assessed using repeated measures two-way ANOVA.


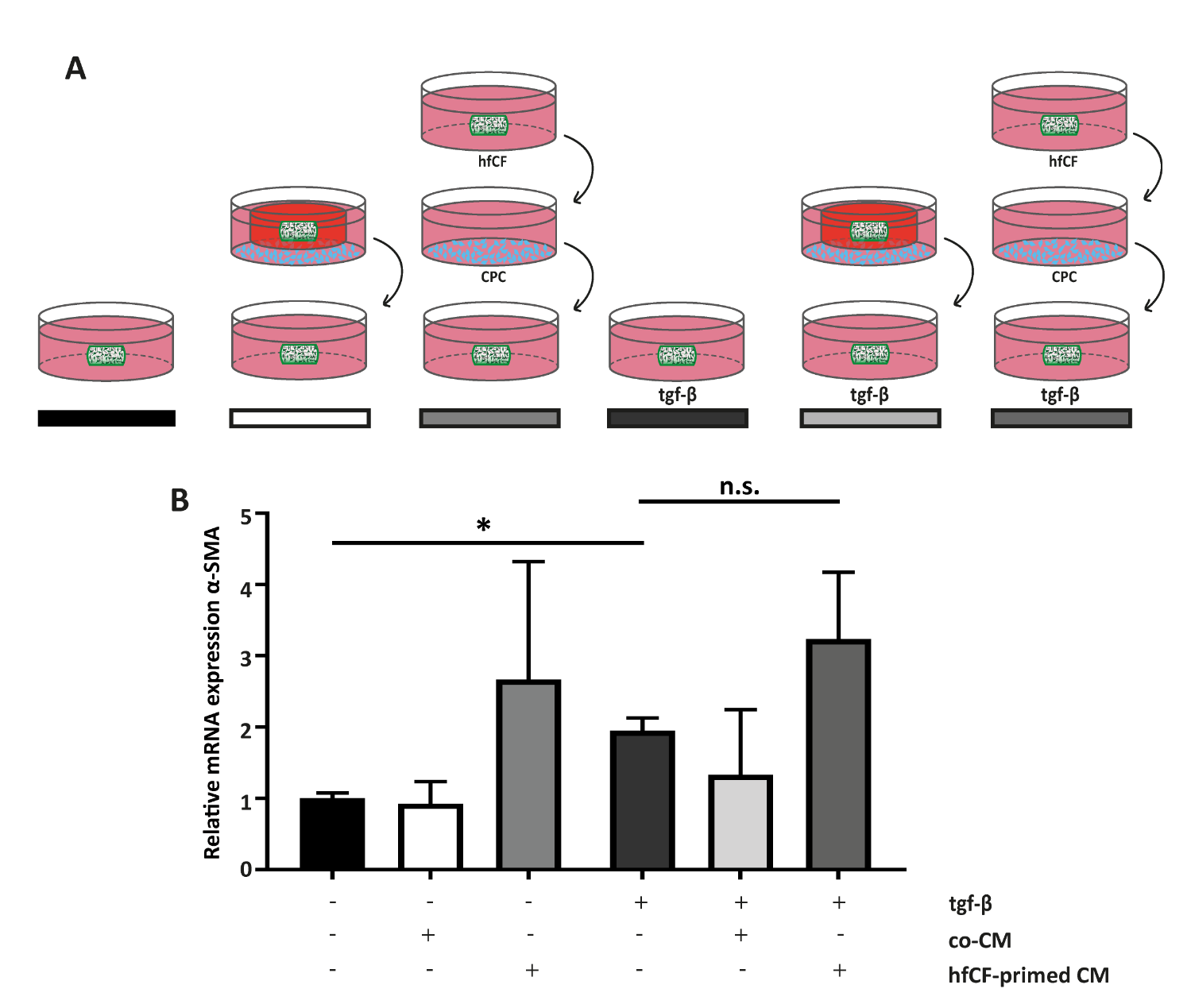


**Supplementary Figure 5. hfCF-primed conditioned medium from cardiac progenitor cells does not inhibit the fibrotic response.**

**A)** Schematic overview of the experimental set-up. **B)** hfCF-primed CM leads to an increase in α-SMA expression in hfCF-laden GelMA in culture conditions with or without 2 ng/ml TGF-β_1_ (n=3). Statistical significance was assessed using repeated measures two-way ANOVA. *p < 0.05.

**Supplementary Table 1. Primers used for quantitative real-time polymerase chain reaction.**

| **Gene** | **Sequence** |
| --- | --- |
| GAPDH | F: 5’-ACAGTCAGCCGCATCTTC-3’ |
|  | R: 5’-GCCCAATACGACCAAATC-3’ |
| α-SMA | F: 5’-AGCCCAGCCAAGCACTG-3’ |
|  | R: 5’-CAAAGCCGGCCTTACAGAG-3’ |
| collagen type 1 alpha chain 1 (COL1a1) | F: 5’-TGCCATCAAAGTCTTCTGC-3’ |
|  | R: 5’-CATACTCGAACYGGAATCCATC-3’ |
